# *N*-(2-Amino-5-chloro­phen­yl)-2-bromo­benzene­sulfonamide

**DOI:** 10.1107/S160053681204562X

**Published:** 2012-11-10

**Authors:** Maria Altamura, Valentina Fedi, Rossano Nannicini, Paola Paoli, Patrizia Rossi

**Affiliations:** a Chemistry Department, Menarini Ricerche S.p.A., Via dei Sette Santi 3, I-50131 Firenze, Italy; b Dipartimento Energetica "Sergio Stecco", University of Firenze, Via S. Marta 3, I-50139 Firenze, Italy

## Abstract

In the title compound, C_12_H_10_BrClN_2_O_2_S, the sulfonamide group adopts a staggered conformation about the N—S bond [the C—S—N—H torsion angle is 97 (3)°] with the N-atom lone pair bis­ecting the O=S=O angle. For the C(Ar)—S bond, the *ortho*-substituted C atom bis­ects one of O=S–N angles [the C—C—S—N torsion angle is −57.7 (3)°]. The mean planes of the aromatic rings form a dihedral angle of 75.1 (1)°. In the crystal, mol­ecules form inversion dimers through pairs of N—H⋯NH_2_ hydrogen bonds. The mol­ecules are further consolidated into layers along the *bc* plane by weaker N—H⋯O inter­actions.

## Related literature

For the synthesis of the title compound, see: Altamura *et al.* (2009 ▶). For the biological activity of sulfa drugs, see: Chegwidden *et al.* (2000 ▶); Lu & Tucker (2007 ▶); Tappe *et al.* (2008 ▶); Purushottamachar *et al.* (2008 ▶). For structural studies of mol­ecules having the sulfonamide –SO_2_—NH group, see: Parkin *et al.* (2008 ▶); Perlovich *et al.* (2009 ▶, 2011 ▶); Vega-Hissi *et al.* (2011 ▶)*;* Altamura *et al.* (2009 ▶, 2012 ▶).
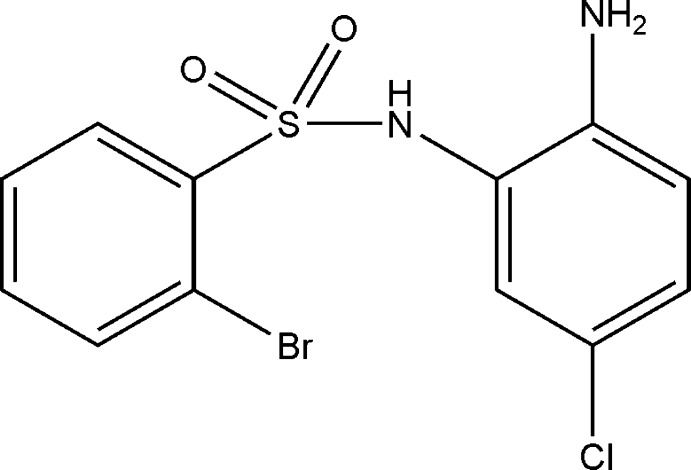


## Experimental

### 

#### Crystal data

C_12_H_10_BrClN_2_O_2_S
*M* *_r_* = 361.64
Monoclinic, 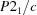

*a* = 13.657 (1) Å
*b* = 14.361 (2) Å
*c* = 7.0829 (9) Å
β = 100.75 (1)°
*V* = 1364.8 (3) Å^3^
*Z* = 4
Mo *K*α radiation
μ = 3.36 mm^−1^
*T* = 298 K
0.32 × 0.26 × 0.22 mm

#### Data collection

Oxford Diffraction Xcalibur3 CCD diffractometer
Absorption correction: multi-scan (*SADABS*; Sheldrick, 1996 ▶) *T*_min_ = 0.365, *T*_max_ = 0.447
6647 measured reflections
2533 independent reflections
1629 reflections with *I* > 2σ(*I*)
*R*_int_ = 0.028

#### Refinement

*R*[*F* ^2^ > 2σ(*F* ^2^)] = 0.038
*wR*(*F* ^2^) = 0.082
*S* = 0.94
2533 reflections
181 parameters
H atoms treated by a mixture of independent and constrained refinement
Δρ_max_ = 0.32 e Å^−3^
Δρ_min_ = −0.38 e Å^−3^

### 

Data collection: *CrysAlis CCD* (Oxford Diffraction, 2006 ▶); cell refinement: *CrysAlis CCD*; data reduction: *CrysAlis RED* (Oxford Diffraction, 2006 ▶); program(s) used to solve structure: *SIR97* (Altomare *et al.*, 1999 ▶); program(s) used to refine structure: *SHELXL97* (Sheldrick, 2008 ▶); molecular graphics: *ORTEP-3* (Farrugia, 1997 ▶); software used to prepare material for publication: *PARST* (Nardelli, 1995 ▶).

## Supplementary Material

Crystal structure: contains datablock(s) I, global. DOI: 10.1107/S160053681204562X/ld2080sup1.cif

Structure factors: contains datablock(s) I. DOI: 10.1107/S160053681204562X/ld2080Isup2.hkl

Supplementary material file. DOI: 10.1107/S160053681204562X/ld2080Isup3.cml

Additional supplementary materials:  crystallographic information; 3D view; checkCIF report

## Figures and Tables

**Table 1:** Selected torsion angles (°)

H*N*1—N1—S1—C1	97 (3)
H*N*1—N1—S1—O1	−19 (3)
C7—N1—S1—O2	50.2 (3)
C6—C1—S1—N1	−57.7 (3)
C6—C1—S1—O1	57.8 (3)

**Table 2:** Hydrogen-bond geometry (Å, °)

*D*—H⋯*A*	*D*—H	H⋯*A*	*D*⋯*A*	*D*—H⋯*A*
N1—H*N*1⋯N2^i^	0.78 (3)	2.26 (3)	3.022 (4)	166 (3)
N2—H*N*2*A*⋯O1^ii^	0.87 (3)	2.45 (3)	3.258 (4)	154 (3)

Symmetry codes: (i) 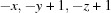; (ii) 
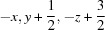
.

**Fig. 1.**
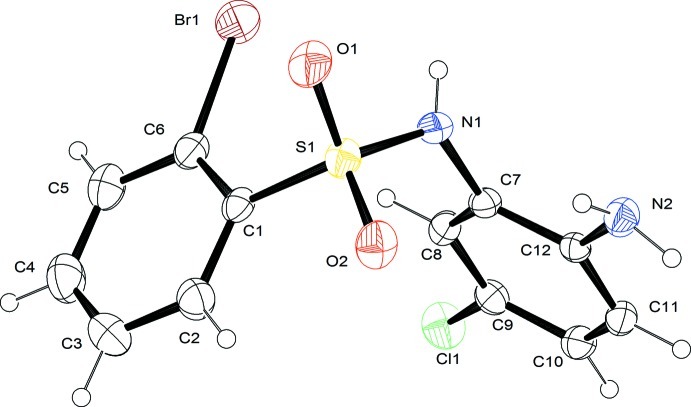
The structure of the title compound showing labelling and displacement ellipsoids drawn at the 30% probability level.

**Fig. 2.**
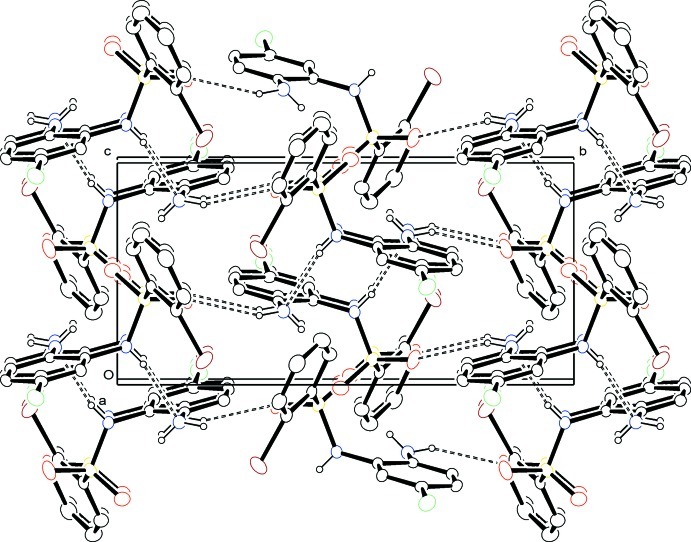
Crystal structure of the title compound as viewed along the a axis. Hydrogen bonds are shown as dashed lines.

## supplementary crystallographic information

### Comment

The study of the structural and conformational properties of the sulfonamide group (R—SO_2_—N*R*_2_) is essential to the comprehension of the "sulfa drugs" action. They found applications as HIV inhibitors (Lu & Tucker, 2007), antimicrobial drugs (Tappe *et al.*, 2008), carbonic anhydrase inhibitors (Chegwidden *et al.*, 2000), anti-tumor agents (Purushottamachar *et al.*, 2008), just to name a few. In this respect a lot of publications have appeared reporting structural data of compounds containing the sulfonamide function (Parkin *et al.*, 2008, Altamura *et al.*, 2009, Perlovich *et al.*, 2009, Perlovich *et al.*, 2011,Vega-Hissi *et al.*, 2011, Altamura *et al.*, 2012). The molecule, as expected, has a staggered conformation about the N—S bond, with the N lone pair bisecting the OŜO angle (Table 1, Fig. 1). The value of the dihedral angle C6—C1—S1—O1 (Table 1) is also in the range observed for arylsulfonamides bearing a non-hydrogen atom in *ortho* position (a bromine atom in this case). The sulfonamide nitrogen atom is almost planar- trigonal (Σ<N=357 (3)°). The aromatic rings are almost perpendicular to each other with a dihedral angle of 75.1 (1)°. In the crystal, dimers are formed by a couple of complementary hydrogen bonds involving the nitrogen atom of the sulfonamide grouping as a donor and amino nitrogen as an acceptor (Table 2). Dimers form layers along bc plane through weaker NH_2_···SO_2_ H-bonds between the amino group and an oxygen atom of the sulfonamide moiety (Table 2). The remaining amine H atom (HN2B) appears to be involved in bifurcated intra-molecular contacts with the oxygen and the nitrogen atoms of the sulfonamide group (HN2B···O2 = 2.72 (3) Å, N2—HN2B···O2 = 132 (3)°; HN2B···N1 = 2.58 (4) Å, N2—HN2B···N1 = 98 (3)°), which could contribute in stabilization of the observed molecular conformation.

### Experimental

For the synthesis of the title compound, see: Altamura *et al.* (2009). Crystals of the title compound suitable for single-crystal X-ray diffraction analysis were obtained by slow evaporation of an ethyl acetate/hexane solution of *N*-(2-amino-5-chlorophenyl)-2-bromobenzenesulfonamide.

### Refinement

The N—H H atoms were located in the Fourier difference map and their coordinates were refined with *U*(H) = 1.2*U*_eq_(N). All other H atoms were positioned using idealized geometry and refined using a riding model with *U*(H) 1.2 times *U*_eq_(C).

### Crystal data

**Table:**

C_12_H_10_BrClN_2_O_2_S	*Z* = 4
*M**_r_* = 361.64	*F*(000) = 720
Monoclinic, *P*2_1_/*c*	*D*_x_ = 1.760 Mg m^−^^3^
*a* = 13.657 (1) Å	Mo *K*α radiation, λ = 0.71069 Å
*b* = 14.361 (2) Å	µ = 3.36 mm^−^^1^
*c* = 7.0829 (9) Å	*T* = 298 K
β = 100.75 (1)°	Prismatic, colourless
*V* = 1364.8 (3) Å^3^	0.32 × 0.26 × 0.22 mm

### Data collection

**Table:**

Oxford Diffraction Xcalibur3 CCD diffractometer	2533 independent reflections
Radiation source: Enhance (Mo) X-ray Source	1629 reflections with *I* > 2σ(*I*)
Graphite monochromator	*R*_int_ = 0.028
Detector resolution: 16.4547 pixels mm^-1^	θ_max_ = 27.3°, θ_min_ = 4.5°
ω scans	*h* = −16→16
Absorption correction: multi-scan (*SADABS*; Sheldrick, 1996)	*k* = −17→18
*T*_min_ = 0.365, *T*_max_ = 0.447	*l* = −8→8
6647 measured reflections	

### Refinement

**Table:**

Refinement on *F*^2^	Primary atom site location: structure-invariant direct methods
Least-squares matrix: full	Secondary atom site location: difference Fourier map
*R*[*F*^2^ > 2σ(*F*^2^)] = 0.038	Hydrogen site location: inferred from neighbouring sites
*wR*(*F*^2^) = 0.082	H atoms treated by a mixture of independent and constrained refinement
*S* = 0.94	*w* = 1/[σ^2^(*F*_o_^2^) + (0.0371*P*)^2^] where *P* = (*F*_o_^2^ + 2*F*_c_^2^)/3
2533 reflections	(Δ/σ)_max_ < 0.001
181 parameters	Δρ_max_ = 0.32 e Å^−^^3^
0 restraints	Δρ_min_ = −0.38 e Å^−^^3^

### Special details

**Table:**

Geometry. All s.u.'s (except the s.u. in the dihedral angle between two l.s. planes) are estimated using the full covariance matrix. The cell s.u.'s are taken into account individually in the estimation of s.u.'s in distances, angles and torsion angles; correlations between s.u.'s in cell parameters are only used when they are defined by crystal symmetry. An approximate (isotropic) treatment of cell s.u.'s is used for estimating s.u.'s involving l.s. planes.
Refinement. Refinement of *F*^2^ against ALL reflections. The weighted *R*-factor *wR* and goodness of fit *S* are based on *F*^2^, conventional *R*-factors *R* are based on *F*, with *F* set to zero for negative *F*^2^. The threshold expression of *F*^2^ > 2σ(*F*^2^) is used only for calculating *R*-factors(gt) *etc*. and is not relevant to the choice of reflections for refinement. *R*-factors based on *F*^2^ are statistically about twice as large as those based on *F*, and *R*- factors based on ALL data will be even larger.

### Fractional atomic coordinates and isotropic or equivalent isotropic displacement parameters (Å^2^)

**Table:**

	*x*	*y*	*z*	*U*_iso_*/*U*_eq_	
O1	0.09409 (17)	0.35073 (16)	0.8889 (4)	0.0597 (7)	
O2	0.11627 (17)	0.51070 (16)	1.0184 (3)	0.0530 (6)	
S1	0.13873 (6)	0.44073 (6)	0.88946 (13)	0.0402 (2)	
Cl1	0.37936 (7)	0.67877 (7)	0.45078 (14)	0.0597 (3)	
Br1	0.26467 (3)	0.30924 (3)	0.61701 (6)	0.0772 (2)	
C1	0.2700 (2)	0.4257 (2)	0.9480 (5)	0.0353 (8)	
C2	0.3192 (3)	0.4646 (2)	1.1197 (5)	0.0501 (9)	
H2	0.2842	0.5022	1.1912	0.060*	
C3	0.4190 (3)	0.4482 (3)	1.1847 (6)	0.0648 (11)	
H3	0.4509	0.4743	1.3000	0.078*	
C4	0.4715 (3)	0.3937 (3)	1.0807 (6)	0.0626 (11)	
H4	0.5389	0.3829	1.1258	0.075*	
C5	0.4252 (3)	0.3548 (2)	0.9097 (6)	0.0527 (10)	
H5	0.4611	0.3183	0.8380	0.063*	
C6	0.3248 (2)	0.3706 (2)	0.8458 (5)	0.0419 (8)	
C7	0.1417 (2)	0.5706 (2)	0.6229 (4)	0.0313 (7)	
C8	0.2332 (2)	0.5803 (2)	0.5710 (4)	0.0362 (8)	
H8	0.2742	0.5286	0.5697	0.043*	
C9	0.2643 (2)	0.6666 (2)	0.5209 (5)	0.0373 (8)	
C10	0.2038 (3)	0.7433 (2)	0.5235 (4)	0.0398 (8)	
H10	0.2252	0.8016	0.4911	0.048*	
C11	0.1118 (2)	0.7335 (2)	0.5742 (4)	0.0391 (8)	
H11	0.0712	0.7854	0.5745	0.047*	
C12	0.0789 (2)	0.6470 (2)	0.6248 (4)	0.0292 (7)	
N1	0.1089 (2)	0.48048 (18)	0.6746 (4)	0.0380 (7)	
HN1	0.091 (2)	0.443 (2)	0.595 (5)	0.046*	
N2	−0.0166 (2)	0.6362 (2)	0.6676 (4)	0.0428 (8)	
HN2A	−0.045 (3)	0.689 (2)	0.687 (5)	0.051*	
HN2B	−0.021 (3)	0.597 (2)	0.750 (5)	0.051*	

### Atomic displacement parameters (Å^2^)

**Table:**

	*U*^11^	*U*^22^	*U*^33^	*U*^12^	*U*^13^	*U*^23^
O1	0.0480 (14)	0.0428 (15)	0.088 (2)	−0.0138 (13)	0.0117 (13)	0.0216 (13)
O2	0.0536 (15)	0.0621 (16)	0.0470 (15)	0.0154 (13)	0.0193 (12)	−0.0013 (12)
S1	0.0373 (5)	0.0376 (5)	0.0473 (5)	−0.0003 (4)	0.0122 (4)	0.0064 (4)
Cl1	0.0463 (6)	0.0695 (7)	0.0655 (7)	−0.0078 (5)	0.0160 (5)	0.0115 (5)
Br1	0.0876 (4)	0.0665 (3)	0.0700 (3)	0.0282 (2)	−0.0049 (2)	−0.0302 (2)
C1	0.0377 (19)	0.0259 (18)	0.043 (2)	−0.0002 (15)	0.0094 (16)	0.0059 (14)
C2	0.053 (2)	0.052 (2)	0.044 (2)	0.008 (2)	0.0050 (18)	−0.0004 (17)
C3	0.062 (3)	0.071 (3)	0.054 (3)	0.001 (2)	−0.009 (2)	−0.006 (2)
C4	0.042 (2)	0.065 (3)	0.077 (3)	0.006 (2)	0.003 (2)	0.012 (2)
C5	0.048 (2)	0.051 (2)	0.062 (3)	0.009 (2)	0.016 (2)	0.0096 (19)
C6	0.044 (2)	0.033 (2)	0.049 (2)	0.0027 (17)	0.0091 (17)	0.0018 (15)
C7	0.0335 (19)	0.0278 (18)	0.0318 (18)	−0.0037 (16)	0.0040 (14)	0.0002 (13)
C8	0.040 (2)	0.0309 (19)	0.038 (2)	0.0035 (16)	0.0072 (15)	0.0028 (14)
C9	0.0369 (19)	0.043 (2)	0.0308 (19)	−0.0014 (17)	0.0046 (14)	0.0013 (14)
C10	0.052 (2)	0.032 (2)	0.034 (2)	−0.0057 (18)	0.0023 (17)	0.0065 (14)
C11	0.048 (2)	0.034 (2)	0.0329 (19)	0.0083 (18)	0.0013 (16)	−0.0009 (14)
C12	0.0305 (18)	0.0313 (18)	0.0250 (17)	0.0009 (16)	0.0029 (13)	−0.0030 (13)
N1	0.0424 (17)	0.0304 (16)	0.0391 (17)	−0.0034 (14)	0.0021 (13)	−0.0018 (12)
N2	0.0429 (19)	0.040 (2)	0.046 (2)	0.0065 (16)	0.0081 (15)	−0.0001 (14)

### Geometric parameters (Å, º)

**Table:**

O1—S1	1.429 (2)	C5—H5	0.9300
O2—S1	1.429 (2)	C7—C8	1.375 (4)
S1—N1	1.605 (3)	C7—C12	1.395 (4)
S1—C1	1.776 (3)	C7—N1	1.438 (4)
Cl1—C9	1.743 (3)	C8—C9	1.377 (4)
Br1—C6	1.892 (3)	C8—H8	0.9300
C1—C6	1.383 (4)	C9—C10	1.379 (4)
C1—C2	1.392 (4)	C10—C11	1.376 (4)
C2—C3	1.375 (5)	C10—H10	0.9300
C2—H2	0.9300	C11—C12	1.390 (4)
C3—C4	1.366 (5)	C11—H11	0.9300
C3—H3	0.9300	C12—N2	1.402 (4)
C4—C5	1.377 (5)	N1—HN1	0.78 (3)
C4—H4	0.9300	N2—HN2A	0.87 (3)
C5—C6	1.380 (4)	N2—HN2B	0.82 (3)
			
O1—S1—O2	119.70 (16)	C8—C7—C12	121.0 (3)
O1—S1—N1	106.66 (16)	C8—C7—N1	120.0 (3)
O2—S1—N1	108.00 (14)	C12—C7—N1	119.0 (3)
O1—S1—C1	107.58 (14)	C7—C8—C9	119.9 (3)
O2—S1—C1	105.30 (15)	C7—C8—H8	120.0
N1—S1—C1	109.34 (15)	C9—C8—H8	120.0
C6—C1—C2	117.8 (3)	C8—C9—C10	120.1 (3)
C6—C1—S1	124.6 (3)	C8—C9—Cl1	120.1 (3)
C2—C1—S1	117.2 (3)	C10—C9—Cl1	119.8 (3)
C3—C2—C1	120.7 (3)	C11—C10—C9	120.0 (3)
C3—C2—H2	119.7	C11—C10—H10	120.0
C1—C2—H2	119.7	C9—C10—H10	120.0
C4—C3—C2	120.4 (4)	C10—C11—C12	120.9 (3)
C4—C3—H3	119.8	C10—C11—H11	119.6
C2—C3—H3	119.8	C12—C11—H11	119.6
C3—C4—C5	120.4 (4)	C11—C12—C7	118.1 (3)
C3—C4—H4	119.8	C11—C12—N2	120.9 (3)
C5—C4—H4	119.8	C7—C12—N2	120.9 (3)
C4—C5—C6	119.1 (3)	C7—N1—S1	121.7 (2)
C4—C5—H5	120.4	C7—N1—HN1	120 (3)
C6—C5—H5	120.4	S1—N1—HN1	115 (3)
C5—C6—C1	121.6 (3)	C12—N2—HN2A	114 (2)
C5—C6—Br1	116.7 (3)	C12—N2—HN2B	116 (3)
C1—C6—Br1	121.6 (2)	HN2A—N2—HN2B	112 (3)
			
HN1—N1—S1—C1	97 (3)	C6—C1—S1—N1	−57.7 (3)
HN1—N1—S1—O1	−19 (3)	C6—C1—S1—O1	57.8 (3)
C7—N1—S1—O2	50.2 (3)		

### Hydrogen-bond geometry (Å, º)

**Table:**

*D*—H···*A*	*D*—H	H···*A*	*D*···*A*	*D*—H···*A*
N1—H*N*1···N2^i^	0.78 (3)	2.26 (3)	3.022 (4)	166 (3)
N2—H*N*2*A*···O1^ii^	0.87 (3)	2.45 (3)	3.258 (4)	154 (3)

Symmetry codes: (i) −*x*, −*y*+1, −*z*+1; (ii) −*x*, *y*+1/2, −*z*+3/2.
